# A neural signature of social support mitigates negative emotion

**DOI:** 10.1038/s41598-023-43273-w

**Published:** 2023-10-12

**Authors:** Rui Pei, Andrea L. Courtney, Ian Ferguson, Connor Brennan, Jamil Zaki

**Affiliations:** 1 Department of Psychology, Stanford University, Stanford, USA; 2 MILA, University of Montreal, Montreal, Canada

**Keywords:** Psychology, Human behaviour, Emotion, Social neuroscience

## Abstract

Social support can mitigate the impact of distressing events. Such stress buffering elicits activity in many brain regions, but it remains unclear (1) whether this activity constitutes a stable brain signature, and (2) whether brain activity can predict buffering across people. Here, we developed a neural signature that predicted social buffering of negative emotion in response to real life stressors. During neuroimaging, participants (n = 95) responded to stressful autobiographical memories either naturally, or by imagining a conversation with a peer. Using supervised dimensionality reduction and machine learning techniques, we identified a spatio-temporal neural signature that distinguished between these two trials. Activation of this signature was associated with less negative affect across trials, and people who most activated the signature reported more supportive social connections and lower loneliness outside the lab. Together, this work provides a behaviorally relevant neurophysiological marker for social support that underlies stress buffering.

## Introduction

Distress is an unavoidable part of life, but thankfully, for most people trouble shared is trouble halved. Support from family, friends, and romantic partners can protect people from the adverse emotional effects of stressful events^1–6^, as well as increase support recipients’ life satisfaction, psychological wellbeing, and physical health^7–11^. Access to social support is associated with reduced experience of physical symptoms such as pain, dizziness, and low energy on high-stress days^12^. When a person’s support system is unavailable, simply imagining contact with supportive others can reduce distress^13,14^.

Social support and interactions constitute important avenues through which individuals satisfy their relationship needs and regulate their emotions^15,16^. When faced with stressful situations, people vary on the type of regulatory strategies (e.g., reappraisal, suppression) they spontaneously rely on to manage their emotions^17^. One factor that contributes to people’s emotion regulation tendencies is social support^6^. For instance, people with higher perceived social support tend to use more reappraisal^18,19^ and less expression suppression strategies^20^. As supportive social interactions may not always be accessible in times of need, the mere presence (either physically or through mental imagination) of a support provider can help individuals buffer negative emotions^21,22^. In addition to social support, empathy is also thought to influence individuals’ emotion regulatory tendencies. Regulating the emotions of ourselves and others’ rely on the capacity the capacity to understand others’ perspectives and feelings^23^. Similar to social support, one’s self-reported capacity to understand others’ emotions is positively related to the habitual use of more adaptive emotion regulation strategies such as reappraisal^24–26^, and negatively related to trait measures of emotion dysregulation^27–29^. More broadly, those who are more empathic and report higher levels of social support are better able to cope with stressors and report higher levels general wellbeing^6,30,31^.

Social support likely helps individuals regulate their emotions through an interplay between the brain’s social cognition network and cognitive control network^32,33^. The cognitive control network encompasses the lateral prefrontal cortex, anterior cingulate cortex, and the parietal cortex. Neural activation in this network is thought to reflect one’s effort to enhance cognitive control of emotion^32^. In addition, the social cognitive network reflects key processes such as mentalizing and empathy, and includes the dorsomedial prefrontal cortex, precuneus, and temporoparietal junction^34,35^. These brain regions could support complex social processes that are important for successful social interactions (e.g., perspective taking)^36,37^ and might reflect processing of prosocial attributes of the interaction partner^38^. Prior studies provide insight on how each brain region or network independently contributes to social support. For example, researchers observed increased activity in the temporo-parietal junction (TPJ), a key region that supports mentalizing and social cognition, when viewing negative pictures after seeing a photo of their friend^32^. However, there is currently no integrative pattern of whole-brain activity that can be used to reliably infer social support using a cross-validation scheme. Cross-validation is essential to establish the validity and generalizability of the neural signature across individuals.

Here, we leveraged machine learning techniques to develop a neural signature for the social buffering of negative emotion. In recent years, machine learning has been increasingly used to help us infer mental states and processes from neuroimaging measurements^39^. These methods have demonstrated promising utility in identifying brain signatures of mental processes such as negative affect^40^, fear^41^, reward^42^ and social perception^43–45^. Extending this approach to social support can help us develop an indicator of how and how well people cope with negative experiences using social resources when they naturally process real-life stressors, which brings both practical and theoretical implications. Practically, a neural signature of social support can provide an objective measure that can be used in the absence of or in addition to self-report measures. For example, as self-report is prone to many biases (for example, subjectivity biases and expectation biases), brain-based predictions may provide biomarkers when evaluating the extent to which people rely on social support while naturally processing stressors. More broadly, an integrative, predictive, and generalizable neural model of social support can help clarify the roles of key psychological processes (e.g., mentalizing, regulation) that underlie social support. This model can also provide insights on the mechanisms through which social support affects wellbeing, which may inform existing theories on the role of social connections in wellbeing^46^.

In this work, we developed and validated a brain signature for social support using a naturalistic task. Participants (n = 95) from a larger social network sample recalled negative autobiographical memories and had 20 s to either focus on the feelings elicited by these memories (self-feel), or imagine a conversation with a peer (imagined support) while placed in a magnetic resonance imaging (MRI) scanner. Our task design made two key advances compared to past studies on social buffering of negative emotion: (1) we used real life, personally relevant stimuli as opposed to aversive images or pain stimuli, and (2) unlike past studies where participants reacted to stimuli in a few seconds, participants in our study spent an extended time period (20 s) to process the stimuli in order to mimic real life experiences.

These design choices allowed our task to mimic emotion regulation experiences outside the laboratory. Given the naturalistic nature of our task, it is important to consider both where and when brain activity associated with support experience occurs. To that end, we used a novel supervised machine learning technique to decode neural time series data into demixed principal components^47^, with each of these components related to a single aspect of the task (e.g. imagined support vs. self-feel). Thus, this method allowed us to extract spatially and temporally relevant latent features that predicted social support in a precise and easy-to-interpret manner. This technique has been used in animal neuronal population data to decode motor behaviors^48^, and similar methods have been used to extract task-relevant low-dimensional components from fMRI time series recordings^49,50^.

We further examined how and whether brain signatures of support tracked individual differences in people’s responses to remembering negative events, and in their social lives outside the lab. To that end, we examined (1) at the trial level, whether expression of the social support signature is associated with less negative affect; and (2) at an individual level, whether expression of this signature is associated with better wellbeing and perceived social support.

## Results

### Social support successfully buffered negative emotion

**Figure 1:**
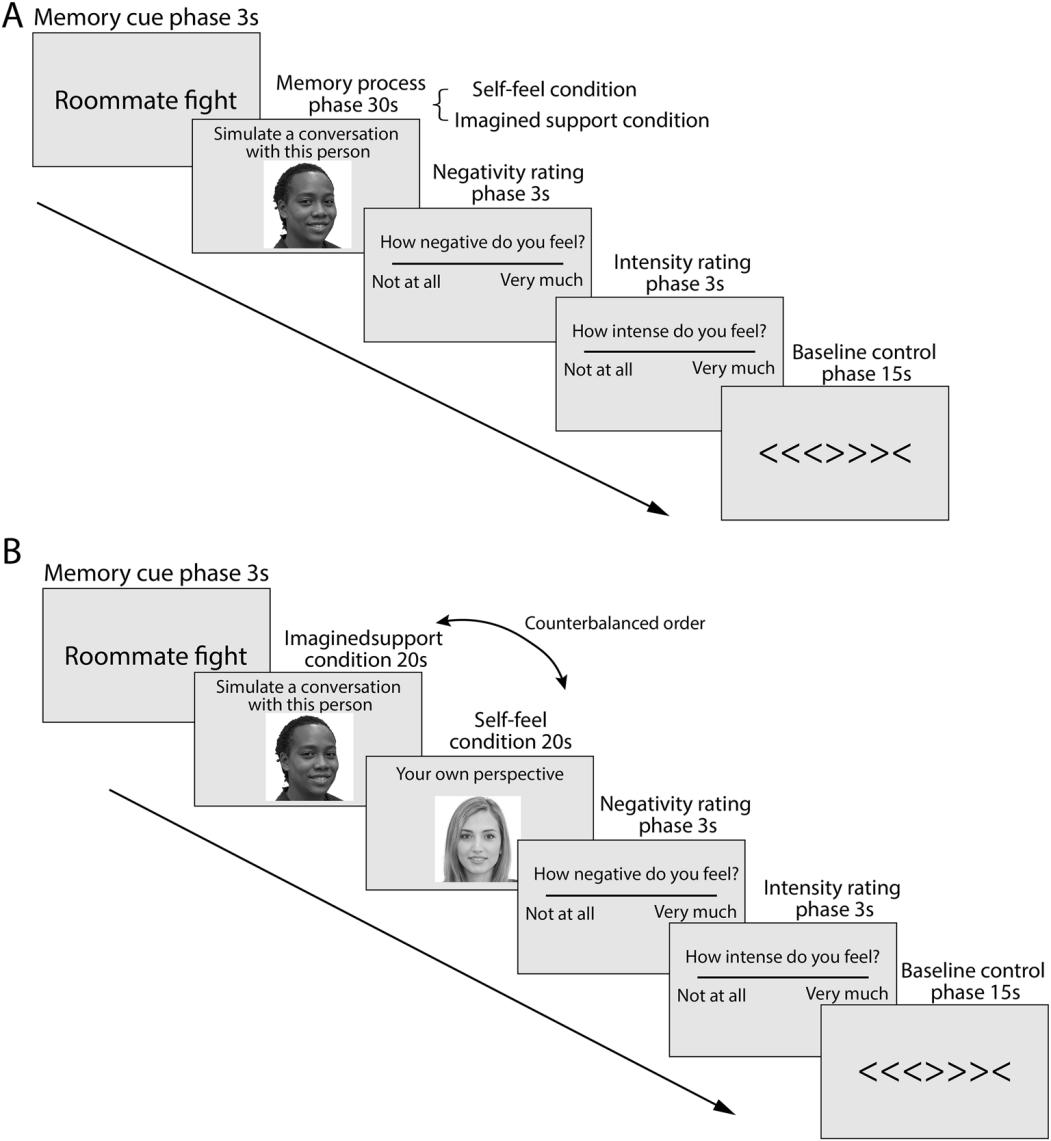
Trial sequence of the fMRI task for (**A**) Sample 1 and (**B**) Sample 2. Note: only the first 20 s of memory process phase in Sample 1 participants were used for decoding to match the trial timings of Sample 2 participants. Photos were computer-generated for illustration purpose only.

Using behavioral data collected from the fMRI task, we first examined whether imagining support from a peer (imagined support condition) would be more effective at buffering negative emotion compared to participants passively experience the feelings elicited by the autobiographical memory (self-feel condition). Our study combined two participant samples that received slightly different versions of the fMRI task (Fig. 1; see “Methods” section for more details). For Sample 1 participants, each memory cue was paired with either imagined support or self-feel condition, allowing us to directly compare the behavioral ratings between the two conditions. For Sample 2 participants, in order to increase the number of trials without burdening the participants to come up with additional memories, each memory cue was paired with both imagined support and self-feel conditions. Sample 2 participants were therefore excluded from this analysis.

Overall, Sample 1 participants rated memories as less negative after imagining a conversation with a dorm mate, compared to experiencing the feelings from their own perspective (M_imagined support_ = 3.93, SD_imagined support_ = 1.82, M_self-feel_ = 4.41, SD_self-feel_ = 1.81, t(282) = − 2.60, p = 0.01). This result indicated that the imagined support condition was more effective at emotion regulation compared to asking participants to focus on the feelings associated with the memories.

In addition to comparing between imagined support and natural process, we also investigated the effectiveness of imagining support as a function of peer type. For both Sample 1 and Sample 2, half of the imagined support trials had a support provider peer as target, and half of the imagined support trials had a non-support provider peer as a target. Support provider peers were defined as dorm members who people would seek support from reported by other dorm members (Sample 1) or by the participants (Sample 2; see “Methods” section). Results showed no significant differences in negativity ratings when imagining support with a support provider vs. a non-support provider. As a result, we collapsed these conditions into an imagined support condition for subsequent analyses.

### A brain signature of social buffering of negative emotion

**Figure 2:**
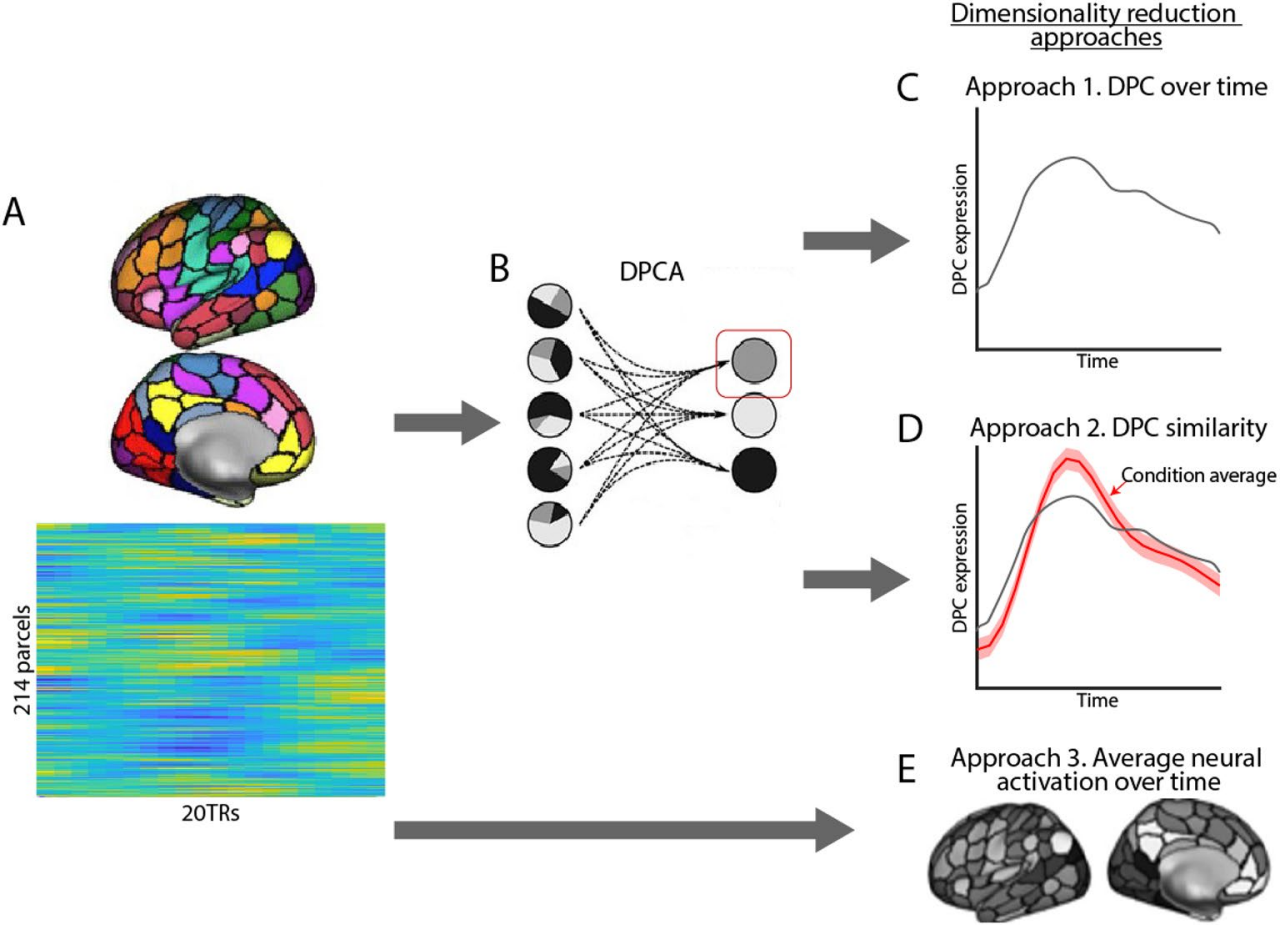
Analyses overview. (**A**) Voxel-level data were first extracted from the whole brain atlas using the 200-parcel cortical parcellation scheme^54^ and 14 subcortical regions using the Harvard–Oxford subcortical atlas^55^. (**B**) Demixed principal component analysis extracts condition-specific principal components. (**C**) For the first approach, the dPC expression over time was used as input feature for the classifier (20 features). (**D**) For the second approach, dPC similarity was calculated by correlating the dPC time series of that trial with the average dPC time series of all trials in the imagined support condition. The dPC similarity was used as input feature for the classifier (1 feature) and mapped onto behavioral measures. (**E**) For the third approach, average neural activity in each of the 214 parcels was used as input for classification.

We next developed a spatially and temporally informed brain signature of social support that distinguished between the imagined support and self-feel conditions. To reduce dimensionality of fMRI time series data, we extracted the mean-response time series from 214 cortical and subcortical regions following prior literature^51^. We then extracted spatial encodings that maximally separate the two conditions using demixed Principal Component Analysis (dPCA; Fig. 2B), which resulted in a temporal trajectory of demixed principal component (dPC) expression for each trial of each participant (Fig. 2C). This temporal trajectory of dPC was used as the feature for cross-validation model prediction.

We applied the extracted dPC time series to train a logistic model to predict the imagined support vs. self-feel contrast in a tenfold cross-validation framework. Test accuracy was assessed by 1000 repetitions of bootstrap testing to determine the significance threshold. In each repetition of the bootstrap, 90% of the total trials were randomly selected to be the training set in which the dPC model was trained on, and the model accuracy was tested on the remaining 10% of the trials. The cross-validation results indicated that whether participant imagined social support can be predicted from the time series of the dPC expression trajectory. The model achieved a reasonable decoding accuracy (out-of-sample test accuracy = 0.69, p_corrected_ < 0.001), which outperformed alternative models using more conventional mean neural activity methods (Supplemental Materials). This decoding accuracy is comparable to other signatures of high-level psychological processes such as autobiographical memory retrieval^52^, as well as decision to receive pain for a future reward^53^.

**Figure 3:**
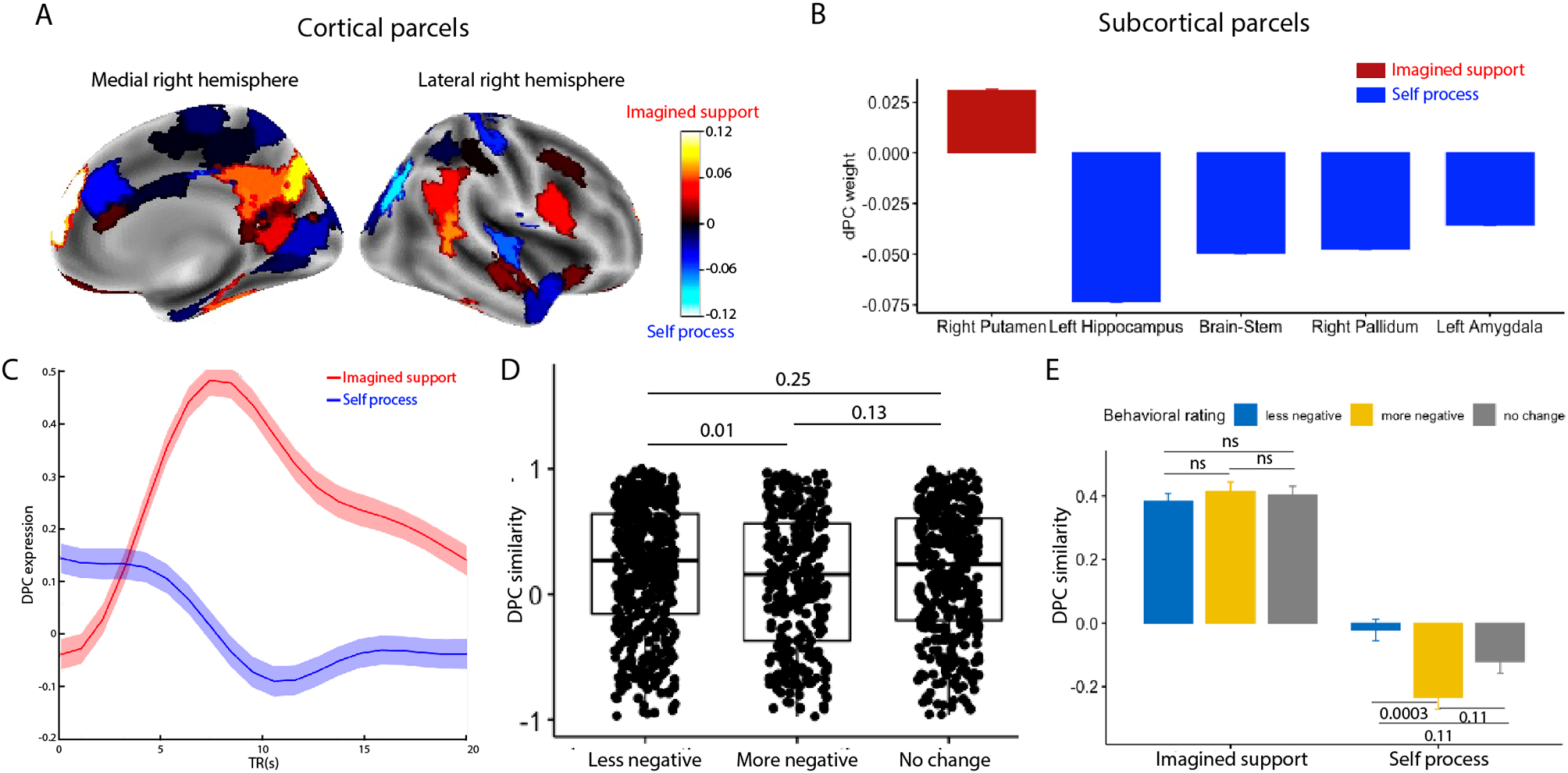
Analyses using the demixed Principal Component Analysis (dPCA; n = 95). (**A**,**B**) Key cortical (**A**) and subcortical (**B**) regions that are robustly identified in condition-specific dPC with bootstrapped 1000 times, FDR corrected p < 0.001. Red clusters indicate regions with positive beta coefficients in the logistic regression (with imagined support being the relevant trial outcome). These regions include dorsomedial prefrontal cortex, precuneus, right fusiform face area, and the right putamen. Blue clusters indicate regions associated with self-feel condition. These regions include supragenual anterior cingulate cortex (sACC), temporal pole, left hippocampus, and left amygdala. (**C**) Condition-specific dPC expression across time in imagined support condition (red) and self-feel condition (blue). (**D**) Trial-level dPC similarity with different behavioral outcomes. Trials with less negative outcomes were significantly higher in dPC similarity compared to trials with more negative outcomes. (**E**) dPC similarity as a function of both condition and behavioral outcomes. The difference observed in (**D**) was driven by trials in the self-feel condition. Error bars indicate standard error, and shaded area in (**C**) indicates 95% confidence interval. P values were FDR adjusted.

We used a parametric bootstrap procedure to identify which parcels most reliably contributed to the classification. Regions that reliably separated imagined support from self-feel trials included DMPFC and precuneus, both key regions in the mentalizing system, as well as the superior frontal gyrus, which was implicated in cognitive control (p_corrected_ < 0.001; Fig. 3A). Regions that are significantly associated with the self-feel condition included supragenual anterior cingulate cortex (sACC) and temporal pole (p_corrected_ < 0.001; Fig. 3A). In addition, the dPCA identified subcortical regions associated with each condition, with the right putamen mapping on to the imagined support condition, and left amygdala mapping on to the self-feel condition (p_corrected_’s < 0.001; Fig. 3B). On the other hand, parcels that least robustly separated the two trial types included regions in the dorsal attention network (including the left superior parietal lobe and the right precentral gyrus) as well as the right hippocampus (see Fig. S3 for more details).

Next, we examined the temporal aspects of emotion regulation by looking at the time course of dPC for each condition (imagined support vs. self-feel; Fig. 3C). Despite the naturalistic nature of our task, dPCA was able to extract a robust time course for both conditions. We observed more temporal variability in dPC time series for the imagined support condition compared to the self-feel condition. Although the separation between the two condition trajectories was significant for most of the trial duration, the magnitude of the separation varied over time. These findings suggest a common temporal dynamic of how people engage the neural signature for trials in each condition despite having idiosyncratic memory stimuli.

### Trial-level signature expression is associated with less negative emotion

We next examined how the expression of this neural signature was related to participants’ negativity ratings at a trial level. Here we focused on dPC similarity, which was operationalized as the Pearson correlation between the dPC expression time course of a trial and the average dPC expression time course of the imagined support condition (Fig. 2D). In other words, dPC similarity represented how much one’s neural dynamics resembled the canonical dynamics of the imagined support condition. We found that trials with less negative ratings were significantly higher in dPC similarity compared to trials with more negative ratings (t(df = 1174) = 2.94, p_adjusted_ = 0.012; Fig. 3D). When further examining dPC similarity as a function of both trial type and behavioral outcome, we found that the difference was driven by distinctions in the self-feel condition, where the dPC similarity for trials in which participants felt more negatively is significantly lower than trials in which participants felt less negatively (t(df = 510) = 4.01, p_adjusted_ = 0.0003; Fig. 3E).These findings indicate that neural similarity to the typical neural response from the imagined support trials can inform emotion regulation outcomes: when people are asked to process memories from their own perspective, the more their neurodynamics resemble those that arise when people imagined supportive others, the less negatively they tend to feel after the trials.

Given that participant’s neural signature expression was correlated with changes in their negative affect rating, we conducted additional analyses to evaluate the specificity and accuracy of our neural signature in representing social support. First, we examined whether our neural signature tracked a general reduction in negative affect instead of simulated support. Using the same cross-validation framework, the neural signature demonstrated a decoding accuracy of 0.57 (7% better than chance level) in predicting reductions of negative affect, which was significantly worse than the decoding accuracy for trial type (0.69; p < 0.001). This finding indicated that the neural signature is specific to decoding imagined support from self-feel trials, rather than negative affect. Second, we compared the performance of our signature with an established neural signature trained on negative affect (PINES)^40^. We found that our model outperformed an alternative model using the PINES signature expression as inputs, which demonstrated that our neural signature was specific for simulated support. See Supplemental Materials for details on these analyses.

### Individual-level signature is associated with better perceived imagined support

**Figure 4:**
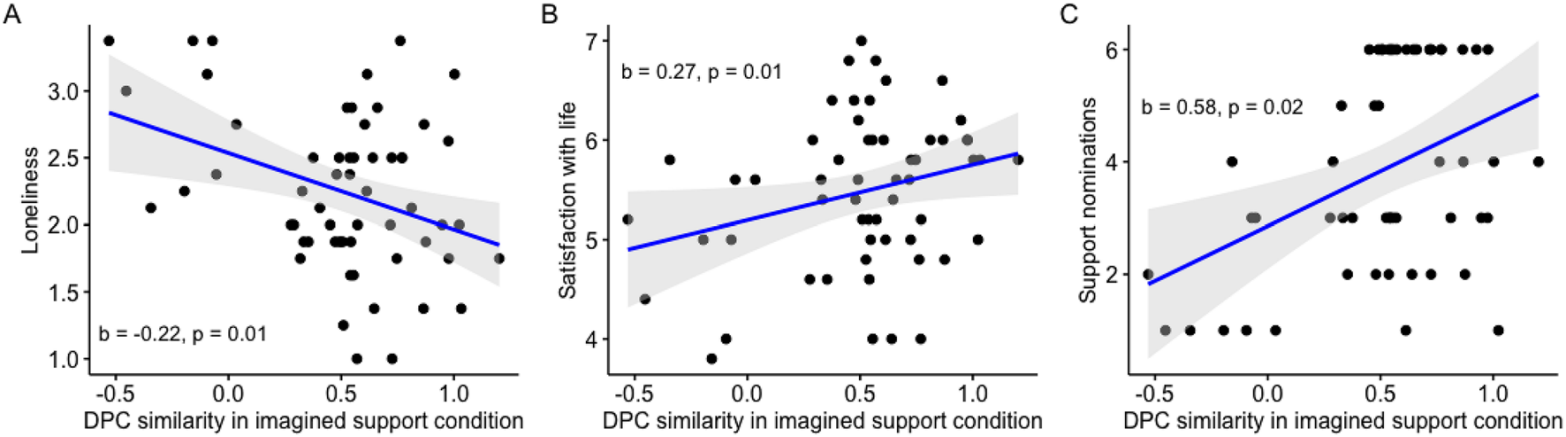
Individual level analyses (n = 95). (**A**) Negative correlation between mean dPC similarity in imagined support condition and loneliness. (**B**) Positive correlation between mean dPC similarity in imagined support condition and satisfaction with life. (**C**) Positive correlation between mean dPC similarity in imagined support condition and perceived social support. Shaded area indicate 95% confidence interval.

We then tested whether this dPC similarity was related to individual difference measures captured prior to fMRI data collection. We theorized that the dPC similarity may indicate the extent to which people can benefit from social support, and that participants’ average dPC similarity in imagined support trials would be associated with higher life satisfaction, lower loneliness, and having more peers that they can turn to for social support. Consistent with our hypothesis, we found that participants with higher dPC similarity during imagined support trials reported lower levels of loneliness (b = − 0.22, 95% CI = [− 0.38, − 0.06], R^2^ = 0.12; Fig. 4A), and higher levels of life satisfaction (b = 0.27, 95% CI = [0.05, 0.48], R^2^ = 0.10; Fig. 4B) after controlling for sample wave and age.

In addition to life satisfaction and loneliness, our results indicated a significant association between dPC similarity and perceived social support. Perceived social support was defined as the number of peers that participants would turn to if something bad happened. On average, participants nominated 3.86 peers that they would turn to with bad news (range = 1–6, SD = 1.81). After controlling for sample wave and age, participants with higher dPC similarity in the imagined support condition reported having more peers that they would share bad news with (b = 0.58, 95% CI = [0.09, 1.08], R^2^ = 0.18; Fig. 4C). Together, the individual-level findings suggest a link between people’s neural responses to imagining social support, their perceived social support, and their wellbeing (i.e., loneliness and satisfaction with life).

## Discussion

Social support is a powerful factor that helps us mitigate the negative effects of stressful life events. Here, we developed and validated a whole brain neural signature of social support that can discriminate between simulating social support and naturally processing a stressor with 69% decoding accuracy in new participants. The weights that contributed most to the model were located in regions that had previously been associated with mentalizing and cognitive control. We further demonstrated that expression of this neural signature was related to behaviorally meaningful indices. Trials with higher expression of this neural signature were likely to be followed by less negative emotion ratings, and people with an overall higher neural signature expression in imagined support trials reported higher life satisfaction, more perceived social support, and less loneliness.

Consistent with prior findings^32^, key regions that contributed to the classification included MPFC (encompassing both ventral and dorsal MPFC), precuneus, TPJ and inferior frontal gyrus. The DMPFC, precuneus, and TPJ are all part of the mentalizing network and have been linked to processes like perspective-taking and emotion regulation^34,56^. In addition, regions that contributed to the imagined support classification included ventrolateral prefrontal cortex and the anterior cingulate cortex, both regions implicated in cognitive control. Although speculative, activity in these regions may indicate participants’ cognitive attempts to change their emotional experiences^33^. On the other hand, regions that least robustly contributed to the separation between two trials included the right hippocampus, left superior parietal lobe and right precentral gyrus. These regions may contribute to general processes related to recalling and processing the autobiographical memories^57^, which is involved in both imagined support and self-feel conditions.

Considering these brain systems holistically and examining their dynamics allowed us to shed new light on the brain mechanisms of support in several ways. First, in expression of this neural support signature maps onto behaviorally relevant indices. At the trial level, when participants were instructed to think about the event from their own perspective, trials with higher signature expression were more likely to be followed by less negative ratings compared to the baseline negativity rating. This finding could indicate that when people are left to their own devices, the more that their neural activity resembles that of social support, the better the emotion regulation outcome. At the individual level, people with higher life satisfaction and perceived social support showed higher signature expression when asked to imagine social support. These findings demonstrate that the neural signature is able to capture aspects of neural processes that are relevant for behavioral outcomes, and provide potential utility of using this neural signature to complement to self-report ratings to evaluate successful emotion regulation of life stressors.

Second, our approach using demixed Principal Component Analysis provided a methodological innovation that allowed us to investigate the temporal dynamics of social support using real-life natural stimuli. Prior studies generally averaged neural activity over a short time period in which people are exposed to aversive images or pain stimuli^3,32,58^. In our results, the model that incorporated both spatial and temporal aspects of neural signals showed better decoding accuracy compared to models that only incorporated spatial neural signals. This suggested that temporal dynamics may play a key role in the regulative benefits of social support. Future studies that explore different task durations (e.g., several seconds, 20–30 s, 5–8 min) can help identify the optimal lengths for effectiveness of simulated support as well as neural prediction.

Although we sought to examine how social support may differ between depending on the target type, we observed no significant differences in change in affect between simulating conversation with a support provider and a non-support provider. There could be several interpretations for this null finding. As participants were asked to imagine conversation with the target in the imagined support condition, the positive effects of this condition could come from the cognitive processes of distancing, perspective taking, emotional expression^59^, or distraction^60,61^, regardless of the target type. An alternative explanation is that our manipulation was not strong enough for our sample size. In the current study, both support providers and non-support providers were students who reside in the same dorm as the participant, with support providers being people whom other students (Sample 1) or the participant (Sample 2) rated as someone from whom they would seek support. Past literature on imagined social support has used attachment figures in comparison to acquaintance as targets^13,62^, or people that participants liked vs. disliked^63^. These manipulations may offer a stronger comparison. Future studies could examine whether simulating support with emotionally close targets (e.g., attachment figures) is more effective at buffering negative emotion compared to simulating support with dorm mates, and whether we may observe a gradient in dPC expression at the neural level that corresponds to the observed behavioral pattern. If supported, these analyses can further add to the validity of the neural signature.

In summary, we developed and validated a neural signature that can differentiate between social support and natural process when people recalled negative autobiographical memories. Expression of this neural signature maps on to behaviorally significant indices such as negative emotion, life satisfaction, loneliness, and perceived social support. These findings highlight the power of social support in buffering negative emotions, and could inform the neural mechanisms through which people benefit from social support. More broadly, an integrative, predictive, and generalizable neural model of social support could review insights on social processes that affect mental health, with implications for people with mental health conditions such as depression and anxiety.

## Methods

### Participants

This study combined participants recruited across two samples as part of a larger study on social connections and well-being in undergraduate students^68^. Participants from both samples were recruited from larger social network samples of first year students of Stanford University. Sample 1 participants were recruited between October 2018 and April 2019 and included forty-eight students (16 females, 16 males, 16 other/prefer not to answer; mean age = 18.24 years, SD = 0.54; 25% Asian, 10% white, 4% black, 8% multiracial, 10% latino/a, and 42% unreported or other race) after excluding three participants due to technical difficulties during scan, and four participants due to excessive movement. Sample 2 participants were recruited between October 2021 and June 2022 and included forty-seven students (26 females, 21 males; mean age = 18.67 years, SD = 0.6; 30% Asian, 13% multiracial, 26% latino/a, 17% white, 2% black, and 13% unreported or other race) after excluding three participants due to technical difficulties during scan, and two participants due to excessive movement. Participants from both samples gave written, informed consent to participate. For both studies, inclusion criteria included first-year students, 18 years or older, fluent in English, and free from MRI contraindications such as no metal in the body, and no surgeries 6 months prior to the MRI scan. The study was approved by the Institutional Review Board at Stanford University, and all methods were carried out in accordance with relevant guidelines and regulations.

### Prescan surveys

Participants from both samples completed two prescan surveys before the fMRI appointment. About 2 to 4 months prior to the fMRI appointment, participants responded to a survey that measured trait variables such as satisfaction with life, loneliness, as well as social network nominations. Participants responded to a second prescan survey within a month prior to the fMRI appointment. In this survey, participants described negative autobiographical memories that were later used in the fMRI task.

#### Social network nomination

Participants as well as other students in their dorms were asked to nominate up to six peers in response to each of the following prompts prior to the fMRI scanning session: “Who do you turn to when something bad happens?”, “Who makes you feel supported and cared for?”, and “Who is the most empathetic?”. Participants’ answers to these questions were used to select target faces in the fMRI Task (see details in “fMRI target selection” section below).

#### Trait measures

Participants reported their life satisfaction using the Satisfaction with Life Scale^64^, and their loneliness levels using the short form UCLA Loneliness Scale (ULS-8)^65^.

#### Autobiographical memory generation

Within a month prior to the scan, participants described nine negative autobiographical memories that would be used later during the fMRI scan. Participants were instructed to describe specific, recent negative experiences (i.e., that had occurred since the beginning of the school year) which still elicited negative emotion. Participants then generated a short title (e.g., “test anxiety”) for each event, and rated the valence (“How negative does this memory make you feel?”) and intensity (“How intense are your emotions when thinking about this event?”) of that memory. Following prior paradigms, the short titles were used to cue their memory for the negative memory during the fMRI task^58^.

### fMRI target selection

We selected dorm mates as emotion regulation targets in both samples. We targeted dorm mates for two reasons. First, dorm mates play a significant role in providing social support, friendship formation, and overall psychological wellbeing for first-year college students as they go through this important transition period^66–68^. For example, college students with more connections to dorm mates are associated with emotion regulation profiles that may be beneficial for well-being, including less reliance on expressive suppression^20^. Second, past work on social buffering of negative emotion has often focused on close friends or romantic partners as emotion regulation targets^2^. We sought to extend this line of work by examining how effective peers—who are less close to the participants, but are nonetheless central to our participants’ social and emotional lives—help participants reduce negative emotion.

In addition to examining whether imagining social support may be an effective emotion regulation strategy, we also sought to investigate how emotion regulation outcomes might differ when imagining support with someone they would vs. would not seek support from. The selection criteria for the targets differed between Sample 1 and Sample 2.

For Sample 1 participants, we used the sociometric measure of indegree centrality in the support network to select support providers vs. non-support providers. Support network was defined using community nominations to the following three questions: “Who do you turn to when something bad happens”, “Who makes you feel supported and cared for?”, and “Who is the most empathetic”. A nomination to at least one of the three questions formed a directed edge in the support network from the nominator to the nominee. Support providers were dorm members whose received nominations were in the top 20% quantile, but did not belong to the personal networks of our participants. Non-support providers were dorm members whose received nominations were in the bottom 20% quantile, who also did not belong to the participants’ personal networks. See Supplemental Materials for indegree centrality statistics of support providers and non-support providers.

Because preliminary analysis in Sample 1 data showed no significant behavioral differences when simulating support with a support provider vs. non-support provider, for Sample 2 participants, we selected imagined support targets using participants’ nominations to the same questions. Support providers were dorm mates who were nominated by the participants in at least one of these questions, and non-support providers were dorm mates who were not nominated by participants in these questions.

Our behavioral analysis showed no significant differences in imagined support with support providers vs. non-support providers for both samples. As such, we collapsed these two conditions into a single imagined support condition in all other analyses.

### fMRI task

The fMRI Task was adapted from methodologies that were used to study how real or imagined presence of loved ones can mitigate brain markers of pain and negative affect^1–3,5,58,69^. Prior to the fMRI scan, participants reviewed the negative autobiographical memory cues to ensure that they could recollect the memory quickly upon viewing the cue. The task consists of four components: memory cue, memory process, emotion rating, and a baseline control task (Fig. 1).

During the task, participants first saw the short mnemonic cue of their negative autobiographical memory that they generated in a prior online survey (memory cue phase). Then, in the memory process phase, they saw a picture of a dorm mate or themselves. When they saw a photo of themselves (self-feel condition), they were instructed to think about the event from their own perspective, focusing on how the event made them feel^69^. When seeing the picture of their dorm mate (imagined support condition), participants were instructed to imagine having a conversation about the cued negative event with the person in the photo. Specifically, they were told to “try to imagine discussing the event with that person. What would they say about what happened? How might talking with them make you feel? Would discussing the event with them make you think about it differently?” After the memory process phase, participants were asked to rate the negativity and intensity of their feelings on a five-point scale (emotion rating phase). Consistent with prior studies on negative biographical memories^58,70^, we focused on participants’ negativity ratings in the main manuscript. See Supplemental Materials for results that relate to intensity ratings. Finally, participants completed a flanker task for the baseline control phase. 

The specific task designs varied between Sample 1 and Sample 2. For participants in Sample 1, this task consisted of three functional runs with counterbalanced orders of three conditions (imagined support with a support provider, imagined support with a non-support provider, and self-feel), with each run lasted 225 s and contained 3, 65-s blocks as well as a 5 s pre-run fixation and a 25 s post-run fixation (Fig. 1A). For Sample 2 participants, we sought to increase the number of memory process trials without burdening participants to come up with additional negative memories. As such, Sample 2 participants completed two functional runs with counterbalanced orders of two conditions (self-feel + imagined support with a support provider, and self-feel + imagined support with a non-support provider; Fig. 1B). The time duration of the memory process phase differed between sample 1 (30 s) and sample 2 (20 s). The time duration of the memory process phase in the fMRI task was based on recent work examining the neural dynamics of social cognitive processes^49,71,72^. We reduced the duration in Sample 2 to allow for more trials with limited scan time. Only the first 20 s of the memory process phase in Sample 1 participants were used for decoding to match the trial timings of Sample 2 participants. For both studies, stimuli were presented using Psychopy^73^ (Study 1: v3.0.1; Study 2: version 2022.1.1).

### Behavioral analyses

We evaluated participants negativity ratings following each memory cue presentation with the following comparisons: (1) imagined support condition (includes both support provider and non-support provider) vs. self-feel condition in Sample 1 participants; and (2) imagined support with a support provider vs. imagined support with a non-support provider in both Sample 1 and Sample 2 participants, respectively. Note that for Sample 2 participants, each block included a self-feel trial and a imagined support trial, we therefore could not compare ratings in the imagined support condition with the self-feel condition in Sample 2 participants.

### fMRI acquisition and preprocessing

fMRI acquisition procedures were the same for Sample 1 and Sample 2 participants. Whole-brain fMRI images were acquired on the same 3.0 Tesla GE Discovery MR750 scanner with a 32-channel head coil. High-resolution anatomical images were acquired during a single scan using a T1-weighted sequence: TR = 7.26 ms, TE = 2.792 ms, flip angle = 12°, pixel bandwidth = 325.55, in-plane resolution (voxel size) = 0.9 mm^3^, slice thickness = 0.9 mm, matrix = 256 × 256, field of view = 23 cm, in-plane acceleration factor = 1.5, GE’s 3D Bravo, interleaved acquisition of 186 axial slices for full brain coverage with no gap. In addition, a spiral fieldmap was acquired to account for inhomogeneities in the magnetic field.

Functional BOLD images were collected with a simultaneous multi-slice (SMS) echo planar imaging (EPI) sequence: TR = 1000 ms, TE = 27 ms, flip angle = 61°, pixel bandwidth = 250, in-plane resolution (voxel size) = 3 mm^3^, slice thickness = 3 mm, matrix = 74 × 74, field of view = 22.2 cm, SMS factor = 4, 48 axial slices acquired (12 mixed slices) for full brain coverage with no gap. Participants viewed the tasks through a mirror mounted at the top of the head coil and made their responses on an fMRI compatible button response box.

MRI data were preprocessed in Nilearn (v0.9.2)^74^ using the fMRIPrep package (v1.0.0-rc5)^75^. The T1-weighted structural images underwent routine preprocessing steps, including skull stripping, segmentation, bias correction, and spatial normalization to the Montreal Neurological Institute (MNI) template. Functional images were realigned and unwarped with reference to the field map and coregistered to the participant’s own structural image.

### Dimensionality reduction

One of the challenges for predictive modeling of fMRI data is the “curse of dimensionality” problem^76^. Since each timepoint of each voxel can theoretically represent a feature, the number of possible features easily exceeds the amount of data points (e.g., the total possible number of features in one trial is 235,375 voxels × 20 TR = 4,707,500 features). As such, dimension reduction and feature selection are likely to be crucial to improve classification results^77^. Here, we adopted a two step supversied machine learning approach to reduce dimensionality. First, we extracted the mean response time series from 214 cortical and subcortical regions following prior literature^51^. Second, we used demixed Principal Component Analysis (dPCA) to find the demixed principal component (dPC) that contained most variance that separated the two conditions (imagined support vs. self-feel).

First, voxel-level data were bandpass filtered between 0.01–0.12 Hz, detrended, standardized, and extracted from the whole brain atlas using the 200-parcel cortical parcellation scheme^54^ and 14 subcortical regions using the Harvard–Oxford subcortical atlas^55^. Nuisance regressors included global signals, anatomical noise components, and non-steady state volume outliers as extracted from the fMRIprep package (v1.0.0-rc5).

Next, we applied dPCA^47^ to extract temporal dynamics that maximally separated between the two conditions. DPCA combines supervised and unsupervised machine learning and breaks down time series data into its individual components. Each of these components relates to only a single aspect of the task and thus allows for easier interpretation. A similar approach has been used in prior work on a theory of mind task^49^, where extracted temporal dynamics of fMRI neural activity during the task were able to classify the trial type in an out-of-sample test set.

For each bootstrap of the cross validation process (see below), we conducted dPCA in the training dataset using MATLAB (The Mathworks Inc., Natick, Massachusetts) scripts available from prior work^47^. Trial condition was entered as the feature to “demix” in the dPCA, and we focused on the extracted demixed principal component (dPC) that contained most amount of condition-specific variance. This resulted in a total of 20 features (1dPC × 20 TRs) that were used for condition classification (Fig. 2C). Thus, dPCA allowed us to reduce dimensionality of neural time series data while losing minimal task-salient information.

We also compared the dPCA dimension reduction method with two alternative approaches. First, we extracted a dPC similarity measure for each trial of each participant by correlating the dPC time series of that trial with the average dPC time series of all trials in the imagined support condition (Fig. 2D). This measure characterizes the extent to which neural activity in a trial resembles the average pattern for the imagined support trials in both spatial and temporal dimensions. This concise measure was used to relate to trial- and individual-level behavioral indices. Second, we used average neural activity in each of the 214 parcels as input for classification (Fig. 2E). This approach resembled common dimensionality reduction techniques used in other work^40,41^, and served as our baseline comparison approach.

### Classification

For each of the dimensionality reduction approach, we trained a logistic regression model to classify imagined support trials from self-feel trials using tenfold cross-validation to estimate the generalizability of this model to new participants. We perform inferences by randomly bootstrapping participants that were included in the training set 1000 times. Because the number of trials in each condition was not the same (56% imagined support trials, 44% self-feel trials), we used a uniform prior to correct for class imbalance in our data. Classification and cross-validation were conducted in Matlab (R2021b). We evaluated the model with balanced F2 test accuracy, sensitivity, specificity, and area under the ROC curve (AUC).

### Linking dPC similarity with self-report measures

We also examined whether dPC similarity was associated with (1) a reduction in negative emotions at the trial level, as well as (2) self-reported social support and wellbeing at the individual level. At the trial level, we constructed mixed-effect regression models to examine whether there were group differences in dPC similarity depending on negativity ratings, after including participant as a random effect. At the individual level, we constructed three ordinary least square (OLS) models to examine the association between individual differences in dPC similarity in imagined support trials and their self-reported loneliness, satisfaction with life, and perceived social support, after controlling for sample wave and age. We corrected for multiple testing with the false discovery rate (FDR) method (Benjamini–Hochberg procedure) in all tested models.

### Supplementary Information

Supplementary Information.

## Data Availability

Behavioral and neuroimaging data, custom code for analyses, and pattern weights from the DPCA are available in an OSF repository: https://osf.io/6qts3/.
