# Supplementary material for: A neural signature of social support mitigates negative emotion
Source: Sci Rep. 2023 Oct 12;13:17293. doi: 10.1038/s41598-023-43273-w (PMC10570303; doi:10.1038/s41598-023-43273-w)
Supplement: Supplementary file 1 — Supplementary Information. [file 41598_2023_43273_MOESM1_ESM.docx]

**Supplementary information**

A neural signature of social support mitigates negative emotion

Rui Pei, Andrea Courtney, Ian Ferguson, Connor Brennan, & Jamil Zaki

**1. Memory process trial instructions**

Prior to the fMRI session, participants were given the following instructions for the memory process phase of the fMRI task. They also completed a brief practice run of the fMRI task before going into the scanner.

*Self-feel trial.* “You will be asked to think about that event. In some cases, accompanying the memory prompt you will see your own photo. When that happens, please think about the event from your own perspective. Consider how it made you feel at the time, and how it makes you feel now. You will have 20s to think about it from your own perspective.”

*Simulated support trial.* “In other cases, accompanying the cue, you will see the face of another person in your dorm. When that happens, try to imagine discussing the event with that person. What would they say about what happened? How might talking with them make you feel? Would discussing the event with them make you think about it differently? You will have 20s.”

Note that for the simulated support condition, we did not explicitly ask participants to imagine a supportive interaction with the dorm mate. Instead, we asked the participants to imagine a conversation and focus on this conversation might make them feel differently about the situation. This design choice was informed by prior work on mere social interactions or presence often serves a regulatory role for people. Similar designs can be found in other work on social support (Eisenberger et al., 2011).

**2. Comparison with alternative models**

To assess the performance of dimension reduction using dPCA model, we compared the performance of the dPCA model with two alternative dimension reduction approaches: (1) dPC similarity and (2) average overtime. In he first comparison approach (“dPC similarity), we correlated the dPC expression time series with the average dPC expression for imagined support trials, resulting in a metric of the similarity of one’s neural activity to that of the typical imagined support trial. This approach resulted in a single feature for cross-validation prediction. The second comparison model wass using average neural activation in each parcel, which resulted in 214 features for cross validation prediction. Classification performance of the three models were shown in Table S1. All models predicted trial type above chance level, with the dPCA model achieving the best decoding accuracy (Two tailed t-test: dPCA vs. dPCA averaged across time: p < 0.005; dPCA vs. mean neural activity: p = 0.02).

**Table S1.** Classification results with different dimensionality reduction approaches.

| Dimensionality reduction approach | # of features | Train accuracy | Test accuracy | AUC | Specificity | Sensitivity |
| --- | --- | --- | --- | --- | --- | --- |
| DPC expression over time | 20 | 0.80 | 0.69 | 0.77 | 0.70 | 0.68 |
| DPC similarity | 1 | 0.75 | 0.65 | 0.71 | 0.65 | 0.65 |
| Mean parcel-level activity | 214 | 0.73 | 0.66 | 0.72 | 0.64 | 0.68 |

**3. Results associated with intensity ratings**

Similar to findings about negativity ratings in the main manuscript, Sample 1 participants rated memories as less intense after imagining a conversation with a dorm mate, compared processing the memories on their own (M_imagined support_ = 3.70, SD_imagined support_ = 1.82, M_self-feel_ = 4.11, SD_self-feel_ = 1.73, t(293) = -2.26, p = 0.02). This result indicated that the imagined support condition was more effective at reducing the intensity of the memory compared to asking participants to naturally process the stimuli. When comparing simulated support with a support provider vs. non-support provider, we found no significant differences in the intensity ratings (t(282) = -0.07, p = 0.95).

We next examined how the expression of this neural signature was related to participants’ intensity ratings at a trial level. We found no significant differences in dPC similarity as a function of changes in intensity rating (ps > 0.05; Fig. S1A). When further examining dPC similarity as a function of both trial type and behavioral outcome, we found marginally higher dPC similarity for trials in which participants felt less negatively vs. trials in which participants felt the same in the self-feel condition, but not simulated support condition (Fig. S1B).

**Figure S1.** Trial-level dPC similarity as a function of (A) changes in intensity ratings, (B) condition and changes in intensity ratings. P values were FDR adjusted.
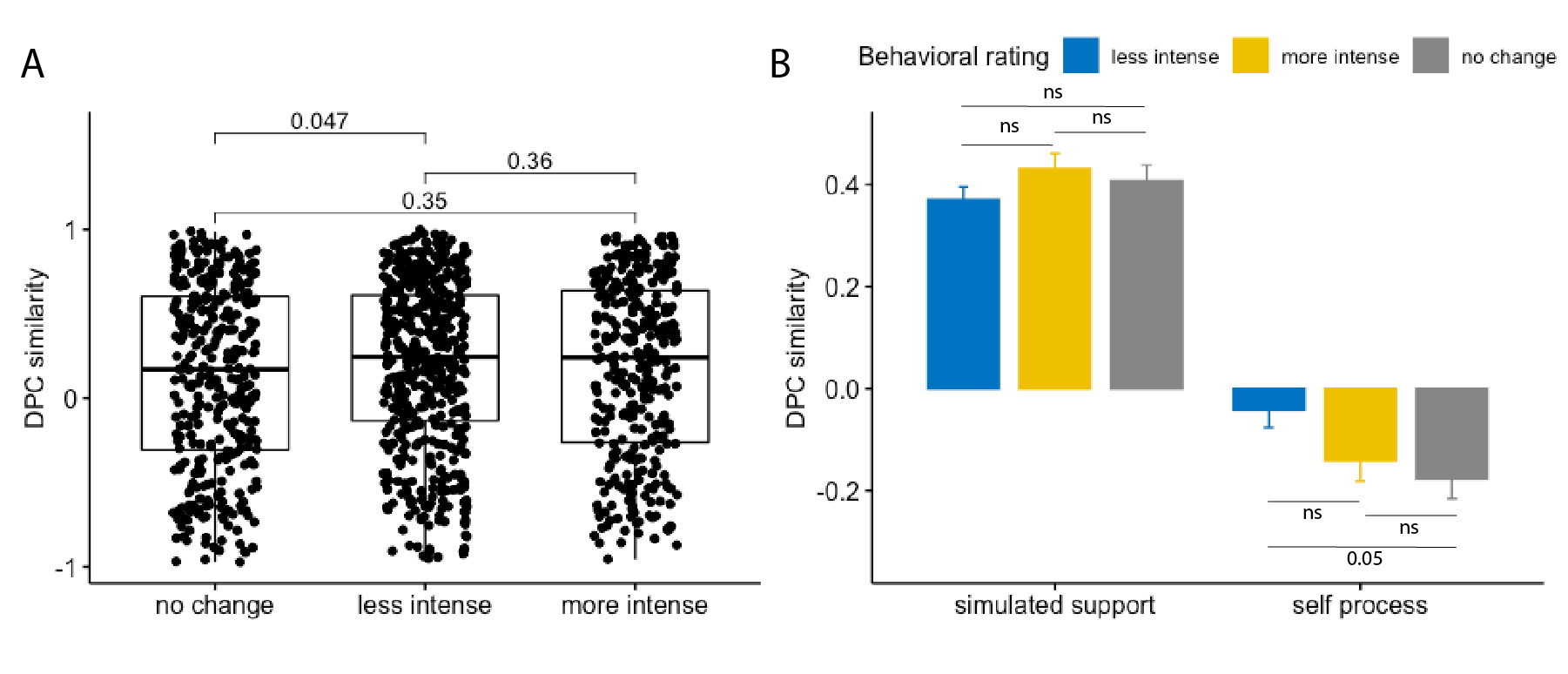


**3. Indegree centrality statistics of simulated support targets.**

**Table S2.** Statistics of empathy network indegree centrality for targets across conditions.

| Target condition | Mean | Median | Range | SD |
| --- | --- | --- | --- | --- |
| Support providers | 2.81 | 3 | [2, 8] | 1.28 |
| Non support providers | 1 | 1 | [1, 1] | 0 |

**Figure S2.** Histogram of empathy network indegree centrality for support providers and non support providers.

**
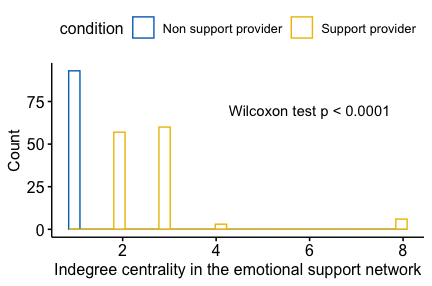
**

**4. Brain parcels that least robustly separated the two conditions**

**Figure S3.** Cortical regions that did not significantly separate the two conditions in the parametric bootstrap analyses.


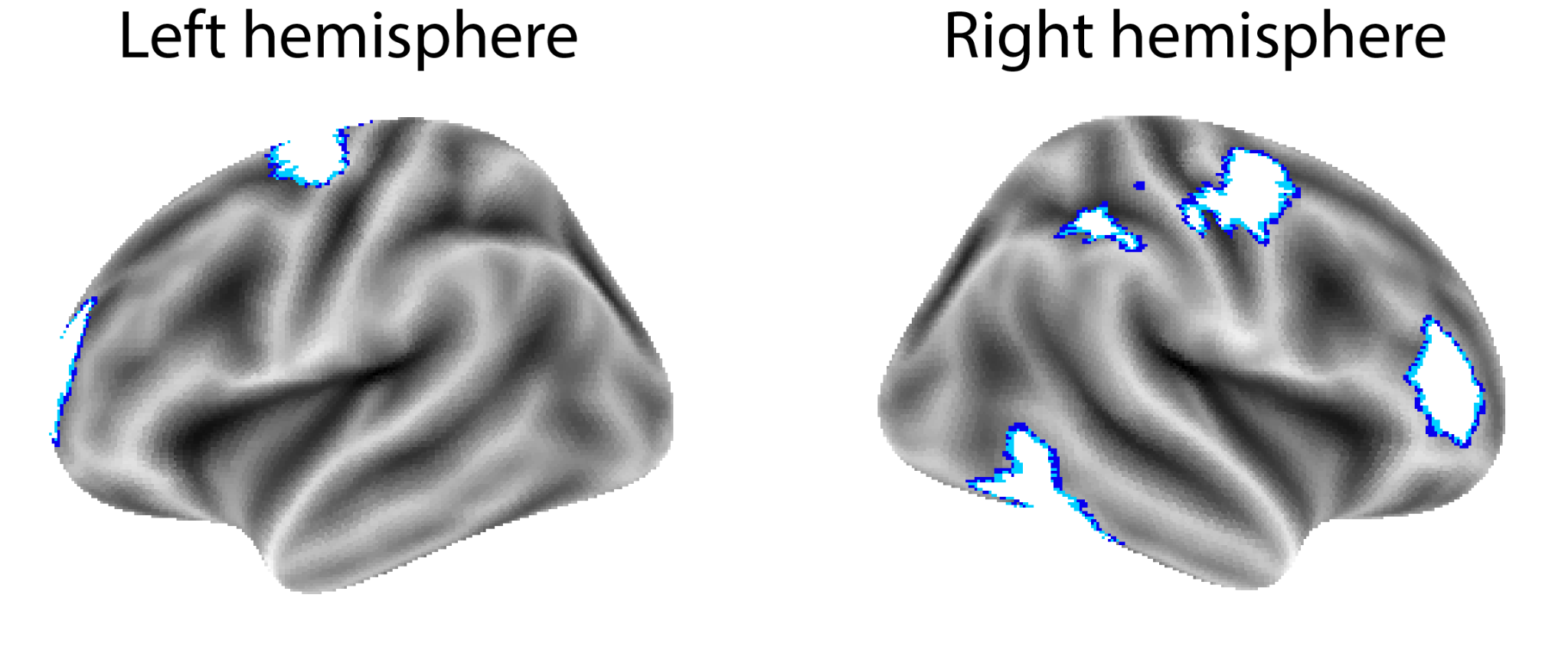


**5. Neural similarity between self-feel and simulated support trials**

We examined wether dPC trajectories of self-feel and simulated support conditions were correlated. On average, neural trajectories for the self- vs. social processing conditions were negatively correlated (r = -0.11, t(70) = -1.9, p = 0.06), indicating a marginal negative correlation between the two conditions at the neural level. This result demonstrated that neural trajectories for the two conditions were sufficiently different from each other.

**6. Model specificity**

We conducted some supplementary analyses to examine whether the developed neural signature reflects simulated social support or a reduction in negative affect in general. First, we examined the prediction accuracy of our neural signature in predicting whether a trial resulted in a reduction in negative affect, regardless of the instructions participants were given during that trial— in essence testing whether our signature tracked feeling better in general. This yielded a decoding accuracy of 0.57 (7% better than chance level), which was significantly worse than the decoding accuracy for trial type (0.69; p < 0.00001). This finding indicated that the neural signature is specific to decoding simulated support from self-feel trials, rather than negative affect.

Second, we compared the decoding accuracy of our neural signature with the PINES negative affect signature (Chang et al., 2015). Overall, PINES expression was significantly higher for the self-feel trial compared to simulated support trial (t(5727) = -5.7, p <0.0001; see Fig. S4). When using trial-level average PINES expression to predict the trial type, the model achieved an overall accuracy of 0.49 (similar to chance level). This finding further supports that the neural signature developed using dPCA outperforms PINES model.

**Figure S4.** PINES signature expression as a function of time and trial type.


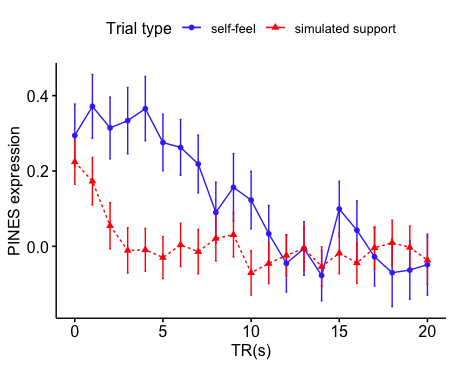


**7. Linking individual level neural similarity and wellbeing**

We also examined whether individual differences in neural similarity between the two trial conditions were correlated with social support and wellbeing. This was evaluated through ordinary least square models with neural similarity between the two trial conditions, sample wave, and age were entered as predictors, and self-reported wellbeing/social support were entered as outcome variable for each model. Results of these analyses indicated that neural similarity between self-feel and simulated support trials was not correlated with (1) loneliness, (2) life satisfaction, nor (3) perceived social support (p’s > 0.05; Table S3).

**Table S3.** Ordinary least squared model results showing the effects of neural similarity between the two conditions at the individual level on outcome variables, controlling for sample wave and age.

|  | Model 1  Loneliness | Model 2  Life satisfaction | Model 3  Perceived social support |
| --- | --- | --- | --- |
| (Intercept) | -0.16 | 0.47 ** | 3.28 *** |
|  | [-0.58, 0.26] | [0.15, 0.79] | [2.43, 4.12] |
| Neural similarity between simulated support and self-feel conditions | 0.02 | 0.04 | -0.00 |
|  | [-0.22, 0.25] | [-0.13, 0.22] | [-0.51, 0.51] |
| Sample wave | 0.22 | -0.09 | 1.24 * |
|  | [-0.30, 0.74] | [-0.48, 0.30] | [0.12, 2.36] |
| Age | -0.22 | 0.08 | -0.06 |
|  | [-0.47, 0.02] | [-0.10, 0.27] | [-0.61, 0.50] |
| R2 | 0.06 | 0.02 | 0.09 |
| All continuous predictors are mean-centered and scaled by 1 standard deviation. *** p < 0.001; ** p < 0.01; * p < 0.05. | | | |

References

Chang, L. J., Gianaros, P. J., Manuck, S. B., Krishnan, A., & Wager, T. D. (2015). A Sensitive and Specific Neural Signature for Picture-Induced Negative Affect. *PLoS Biology*, *13*(6), e1002180. https://doi.org/10.1371/journal.pbio.1002180

Eisenberger, N. I., Master, S. L., Inagaki, T. K., Taylor, S. E., Shirinyan, D., Lieberman, M. D., & Naliboff, B. D. (2011). Attachment figures activate a safety signal-related neural region and reduce pain experience. *Proceedings of the National Academy of Sciences of the United States of America*, *108*(28), 11721–11726. https://doi.org/10.1073/pnas.1108239108
